# No-reference image quality assessment for confocal endoscopy images with perceptual local descriptor

**DOI:** 10.1117/1.JBO.27.5.056503

**Published:** 2022-05-18

**Authors:** Xiangjiang Dong, Ling Fu, Qian Liu

**Affiliations:** a Huazhong University of Science and Technology, Wuhan National Laboratory for Optoelectronics, Wuhan, China; b Hainan University, School of Biomedical Engineering, Key Laboratory of Biomedical Engineering of Hainan Province, Hainan, China

**Keywords:** confocal endoscopy, image quality assessment, local binary pattern, differential excitation, human visual system

## Abstract

**Significance:**

Confocal endoscopy images often suffer distortions, resulting in image quality degradation and information loss, increasing the difficulty of diagnosis and even leading to misdiagnosis. It is important to assess image quality and filter images with low diagnostic value before diagnosis.

**Aim:**

We propose a no-reference image quality assessment (IQA) method for confocal endoscopy images based on Weber’s law and local descriptors. The proposed method can detect the severity of image degradation by capturing the perceptual structure of an image.

**Approach:**

We created a new dataset of 642 confocal endoscopy images to validate the performance of the proposed method. We then conducted extensive experiments to compare the accuracy and speed of the proposed method with other state-of-the-art IQA methods.

**Results:**

Experimental results demonstrate that the proposed method achieved an SROCC of 0.85 and outperformed other IQA methods.

**Conclusions:**

Given its high consistency in subjective quality assessment, the proposed method can screen high-quality images in practical applications and contribute to diagnosis.

## 1 Introduction

Confocal endoscopy employs laser scanning confocal imaging technology to achieve real-time observation of mucosal cells and subcellular structures with micron-scale resolution to accurately locate lesions.^1^^,^^2^ Probe-based confocal endoscopes are commonly used. They transmit laser and fluorescence images via a fiber bundle,^3^ which has flexibility and accessibility for *in vivo* clinical imaging. The miniature objective system is the core component of confocal endoscopy for high resolution to directly visualize cells and is often assembled from multiple optical elements to correct aberrations.^4^ It improves biopsy accuracy and contributes to the early diagnosis of cancer in various clinical fields such as human brain tumors^5^ and gastrointestinal cancer.^6^

However, distortions, such as blur, noise, and decreased contrast, are common in confocal endoscopy imaging. Blur is the most common type of distortion in confocal endoscopy, which is caused by defocus, probe fiber core cross coupling,^7^ and motion caused by the difference in movement speed between the investigated anatomical structures and the physician.^8^ Owing to the small field-of-view of the confocal endoscopy,^9^ to obtain a comprehensive view, a typical endoscopy examination produces thousands of images, most of which are not useful for diagnostic purposes^5^ owing to image degradation and information loss caused by the above distortions. Manual removal of nondiagnostic and low-quality images is time-consuming and labor-intensive. Therefore, it is desirable to automatically screen high-quality images accurately and efficiently. An image enhancement method is observed to be beneficial in the automatic diagnosis of confocal endoscopy images,^10^ and its development and evaluation also require the involvement of image quality assessment (IQA). Furthermore, the imaging performance evaluation of the confocal endoscopy also requires the participation of IQA; for example, Wang et al. analyzed the image histogram distribution to assess image contrast to validate the proposed confocal microendoscope.^11^ Therefore, it is essential to develop an IQA method because it can benefit clinical applications of confocal endoscopy.

IQA is mainly divided into full reference (FR) and no reference (NR) methods. FR-IQA requires an ideal undistorted image when evaluating the quality, which is difficult to obtain in practical applications. NR-IQA is gaining attention because it does not require the use of reference images. The feature extraction and prediction model form the general NR-IQA framework. Common features describe poetry of images, including natural scene statistics (NSS) feature,^12^^,^^13^ gradient feature,^14^ frequency domain feature,^15^ curvelet domain feature,^16^ and discrete cosine transform (DCT) domain feature.^17^ In addition, there are methods based on analysis of the perceptual process of image reception by the human eye, that is the human vision system (HVS), such as free energy theory^18^ and phase congruency.^19^ Owing to the powerful ability of image description, local descriptors in IQA have aroused extensive attention, such as local binary pattern (LBP),^20^^,^^21^ from accelerated segment test (FAST),^22^ speeded-up robust features (SURF),^23^ Weber local descriptor (WLD),^24^ and they have remarkable performance in multiply-distorted images.

Currently, NR-IQA has promising performance for images with a single distortion, such as blur, noise, and JPEG compression, while it is unsatisfactory when it comes to authentic and multiply distorted images^25^ owing to joint distortion interactions. Deep learning techniques for IQA have been studied; however, the size of the dataset limits the network structure.^26^ Bianco et al.^27^ proposed the DeepBIQ method that uses a fine-tuned convolutional neural network (CNN) to exact features and feeds it to support vector regression (SVR) to predict the image quality. Liu et al.^28^ proposed RankIQA, first pretrained the network on a large-scale self-build image pair database for an quality comparison of image pair task and fine-tuned the network to achieve promising performance. Ma et al.^29^ proposed MEON, which first pretrained the network by an image distortion classification task on synthetic distortion images and then fine-tuned the network to achieve end-to-end image quality prediction. Zhu et al.^30^ proposed MetaIQA method, first adopted the meta-learning strategy to learn the prior knowledge from different NR-IQA tasks and fine-tuned the network to address the small sample problem. In general, deep learning requires a great number of training images, and the scale of the image quality database severely limits the performance of deep networks. Establishing larger datasets and designing a new method to reduce the requirement for training images is a direction that needs further exploration for deep IQA.

Medical images are multiply distorted because of variable imaging conditions; meanwhile, the content of medical images is distinct from that of natural images, mainstreaming IQA may decline in performance.^31^ It is necessary to develop IQA of medical images. The study of medical IQA has increased because of the development of deep learning techniques, and it mainly focuses on ultrasound imaging, MRI, and OCT images. Zhang et al.^32^ proposed DCNN-IQA-14 and ResNet-IQA based on traditional networks to predict the quality of ultrasound images. To overcome overfitting, a transfer-learning strategy was employed. Liu et al.^33^ proposed a nonlocal residual neural network to assess slicewise MRI image quality and applied random forest for the volumewise quality grade. Semisupervised learning and iterative self-training strategies were used for a few quality-annotated images. Wang et al.^34^ analyzed the performance of four classic deep networks for assessing the quality of retinal OCT images by transfer learning, and the ResNet-50 network achieves highest performance. Medical images are more difficult to acquire and label than natural images, making the deep learning IQA method difficult to develop, transfer learning strategies based on traditional networks are widely adopted, and the potential of deep learning has yet to be fully explored.

The analysis of confocal image quality has made some progress. Aubreville et al.^8^ proposed an improved Inception v3 network to detect motion artifacts in confocal endoscopy images. Kamen et al.^35^ screened high quality and information-rich images by calculating image entropy before classifying images. Izadyyazdanabadi et al.^36^ proposed a binary classification network to classify diagnostic and nondiagnostic images and applied fine-tuning and ensemble modeling techniques to improve performance and achieve high accuracy.^37^ Despite this, none of these methods can quantify image quality, which limits the applications for screening and evaluating image enhancement algorithms. The signal-to-noise ratio is determined by a confocal endoscopy IQA^38^ that can quantify image quality. However, regions of interest need to be manually selected before calculation, which does not apply to practical applications.

To meet the needs of practical applications, in this paper, we propose a new NR confocal laser endoscopy IQA (CEIQA) method, which combines local descriptors and Weber’s law. First, we used a differential excitation (DE) map to describe the local variation information, calculated the LBP map of the image to describe structure information, and then computed the joint distribution histogram of DE and LBP as the first feature set. Second, for better perception of the ability to describe image information, we improved local ternary pattern (LTP) by changing its threshold function by referring to Weber’s law and computed the histogram and entropy of improved LTP, which was used to measure the distribution of local variation patterns. Finally, SVR was applied to map the perceptual features to the quality score. Our main contributions are summarized as follows:

1. An NR-IQA method using local descriptors for confocal endoscopy images is proposed. The relationship between the local descriptors and image quality is detailed and analyzed.
2. A new dataset containing 642 confocal endoscopy images with corresponding subjective mean opinion score (MOS) from eight experienced researchers is established.
3. An extensive evaluation was conducted for the proposed method and other state-of-the-art NR-IQA methods. The experimental results show that CEIQA significantly outperforms other state-of-the-art methods.

The remainder of this paper proceeds as follows. In Sec. 2, we present the feature-extraction method for the proposed IQA. In Sec. 3, we present the details and results of the performance experiments. In Sec. 4, we discuss the limitations of the study and future work. Finally, in Sec. 5, we conclude the main contributions and provide potential application prospects for the proposed IQA.

## 2 Materials and Methods

### 2.1 Weber’s Law and Differential Excitation

**Fig. 1:**
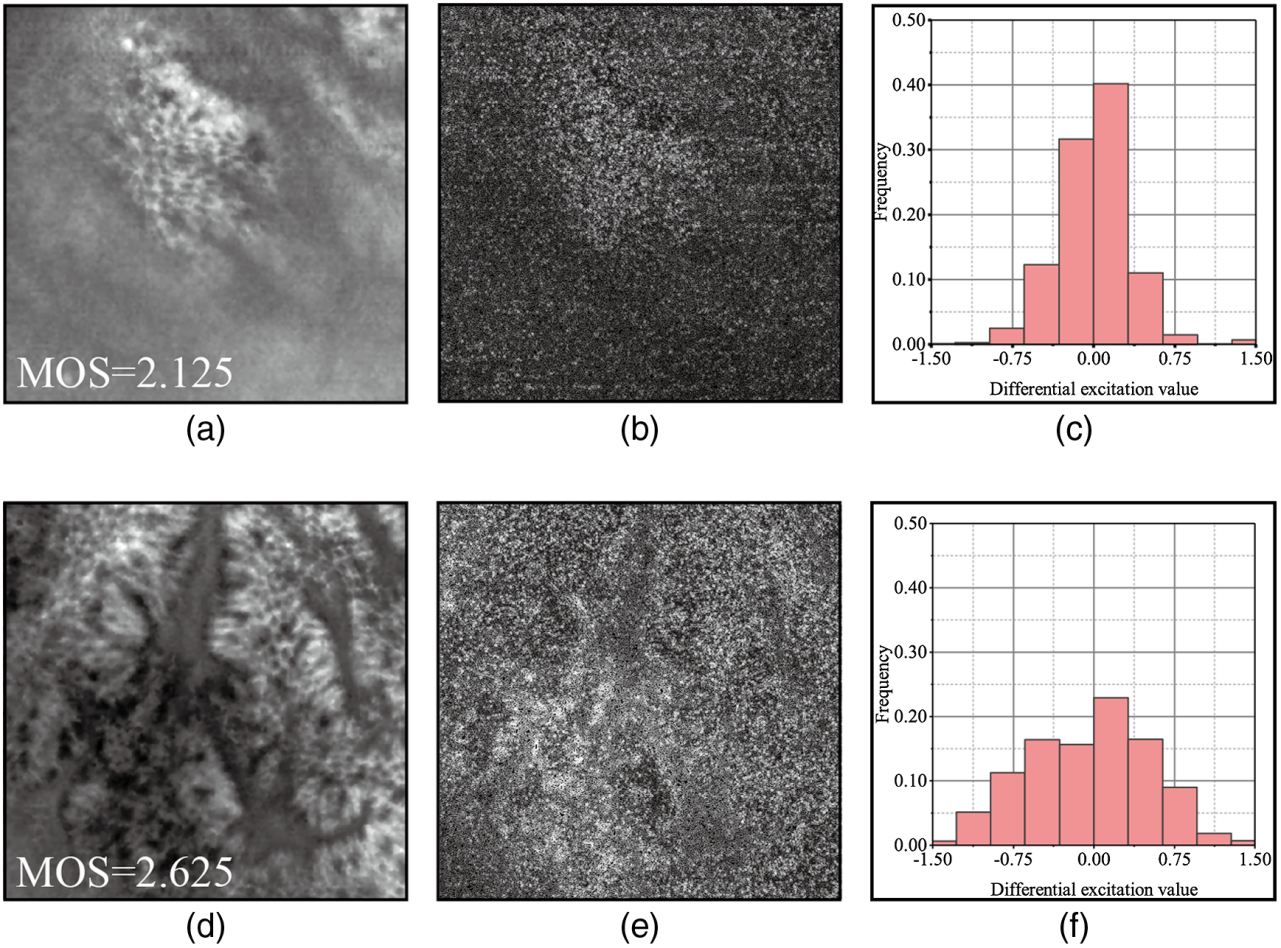
DE of confocal endoscopy images. (a) and (d) Image with MOS of 2.125 and 2.625, respectively. (b) and (e) Corresponding DE map. DE map value is turned to 0−π/2 by taking the absolute value and is scaled to 0 to 255. (c) and (f) Frequency histogram of DE map.

The perceptual process is sensitive to relative variations in pixel intensity in images when recording to the HVS. Weber’s law can be used to describe this mechanism,^39^ which is expressed as follows:

$$\frac{\Delta I_{\mathrm{th}}}{I}=k,$$ (1)

where $\Delta I_{\mathrm{th}}$ represents the perceptual threshold, I represents the initial stimulus intensity, and k is a constant. Based on Weber’s law, Chen et al.^40^ proposed the WLD to extract local variation information in the image. One of the components is DE, which is calculated as follows:

$$I_{\mathrm{DE}}=\arctan(\frac{\Delta I}{I})=\arctan(\overset{p-1}{\underset{i=1}{\sum }}\frac{x_{i}-x_{c}}{x_{c}}),$$ (2)

where I denotes the original image, ΔI characterizes the image local variation, $I_{DE}$ is the DE map, $x_{c}$ is the central pixel, $x_{i}$ is the neighborhood pixels, p is the number of neighborhood pixels, and arctan function is used to prevent computation instability. After the above calculation, the range of $I_{\mathrm{DE}}$ becomes [−π/2,π/2]. Compared to Weber’s law, DE regards the sum of the difference between the neighbors and the center as the change in the image. In this study, we adopted DE to describe the local variation in confocal endoscopy images. Figures 1(a) and 1(d) show two confocal endoscopy images with different MOSs and DE maps, respectively.

Figures 1(b) and 1(e) show that DE highlights the variation region in the image; thus, the distribution of the DE values shown in Figs. 1(c) and 1(f) represents the distribution of levels of local variation in the image. A low variation region means “the expected” according to the free energy theory,^18^ which carries less information than a high-variation region. Figures 1(c) and 1(f) demonstrate that the DE value of the low-quality image is more often located around the zero point, whereas that of the high-quality image is more evenly distributed. In conclusion, the distributional characteristics of DE can be used as perceptual features that indicate image quality.

Nonetheless, Eq. (2) shows that during the accumulation phase, positive and negative variations will counteract each other, resulting in image variation information loss; meanwhile, the pattern and direction of local variation are ignored. Therefore, further analysis based on a DE map is required.

### 2.2 Improved LBP by Differential Excitation

The LBP^41^ is a local descriptor with remarkable performance and has attracted considerable attention in IQA studies^20^^,^^21^ owing to its ability to describe the local structure of an image. By comparing the interpixel relationships between the central pixel and its neighbors, LBP divides pixels into different patterns. The general form of LBP is rotation-invariant uniform $\mathrm{LBP}({\mathrm{LBP}}^{\mathrm{riu}2})$,^41^ defined as follows:

$${\mathrm{LBP}}_{P,R}^{\mathrm{riu}2}=\{\begin{array}{ll} \overset{P-1}{\underset{i=0}{\sum }}S(g_{i}-g_{c}), & u({\mathrm{LBP}}_{P,R})\leq 2\\ P+1, & \text{else} \end{array},$$ (3)

where $g_{c}$ and $g_{i}$ are the central and circular neighborhood pixels, respectively, R is the radius of circular neighborhood, P is the number of $g_{i}$, and S(·) is the thresholding function defined as follows:

$$S(g_{i}-g_{c})=\{\begin{array}{cc} 1, & g_{i}-g_{c}\geq 0\\ 0, & g_{i}-g_{c}<0 \end{array},$$ (4)

where u(·) defined in Eq. (5) is a bitwise transition function that recognizes a uniform pattern whose number of bitwise transitions is less than two. Uniform patterns contribute more to the description of the image structure than basic patterns. ${\mathrm{LBP}}^{\mathrm{riu}2}$ has P+1 uniform patterns and one other pattern, coding from 0 to P+1:

$$u({\mathrm{LBP}}_{P,R})=\|S(g_{P-1}-g_{c})-S(g_{0}-g_{c})\|+\overset{P-1}{\underset{i=0}{\sum }}\|S(g_{i}-g_{c})-S(g_{i-1}-g_{c})\|.$$ (5)

The image structure contains information, and quality degradation will affect it, resulting in pattern shifts in the LBP.^20^ Therefore, LBP can be used in the representation of image quality. The obtained LBP map is defined as follows:

$$I_{\mathrm{LBP}}={\mathrm{LBP}}_{P,R}^{\mathrm{riu}2}(I).$$ (6)

DE only calculates the intensity information and ignores local variation information of pattern and direction; meanwhile, LBP contains interpixel relationships without pixel intensity information. Therefore, it is helpful to calculate the joint distribution histogram of DE and LBP to compensate for both the shortage in describing the structure and preserving the local variation intensity and pattern of pixels. The joint distribution histogram was calculated as follows:

$$H(m,n)=P(I_{\mathrm{LBP}}=m\cap I_{\mathrm{DE}}=n),$$ (7)

where H(m,n) is a two-dimension joint histogram, P(·) indicates the frequency function. m∈{0,…,M}, where M=P+1 is the number of uniform patterns of $I_{\mathrm{LBP}}$ and n∈{1,…,N}, where N is the number of bins in the histogram of the $I_{\mathrm{DE}}$. After the joint histogram H(m,n) is obtained, it is an M×N dimension feature to characterize the local variation and structure of the image.

### 2.3 Improved LTP by Weber’s Law

The LTP^42^ is the generalization of LBP by changing the threshold function to obtain more detailed information of the local interpixel relationship. LTP was calculated as follows:

$$\mathrm{LTP}=\overset{P-1}{\underset{i=0}{\sum }}3^{i}\cdot T(g_{i}-g_{c}),$$ (8)

$$T(g_{i}-g_{c})=\{\begin{array}{ll} 1, & g_{i}-g_{c}>t\\ 0, & |g_{i}-g_{c}|\leq t\\ -1, & g_{i}-g_{c}<-t \end{array},$$ (9)

$${\mathrm{LTP}}_{\text{up}/\text{low}}=\overset{P-1}{\underset{i=0}{\sum }}2^{i}C_{\text{up}/\text{low}}.$$ (10)

**Fig. 2:**
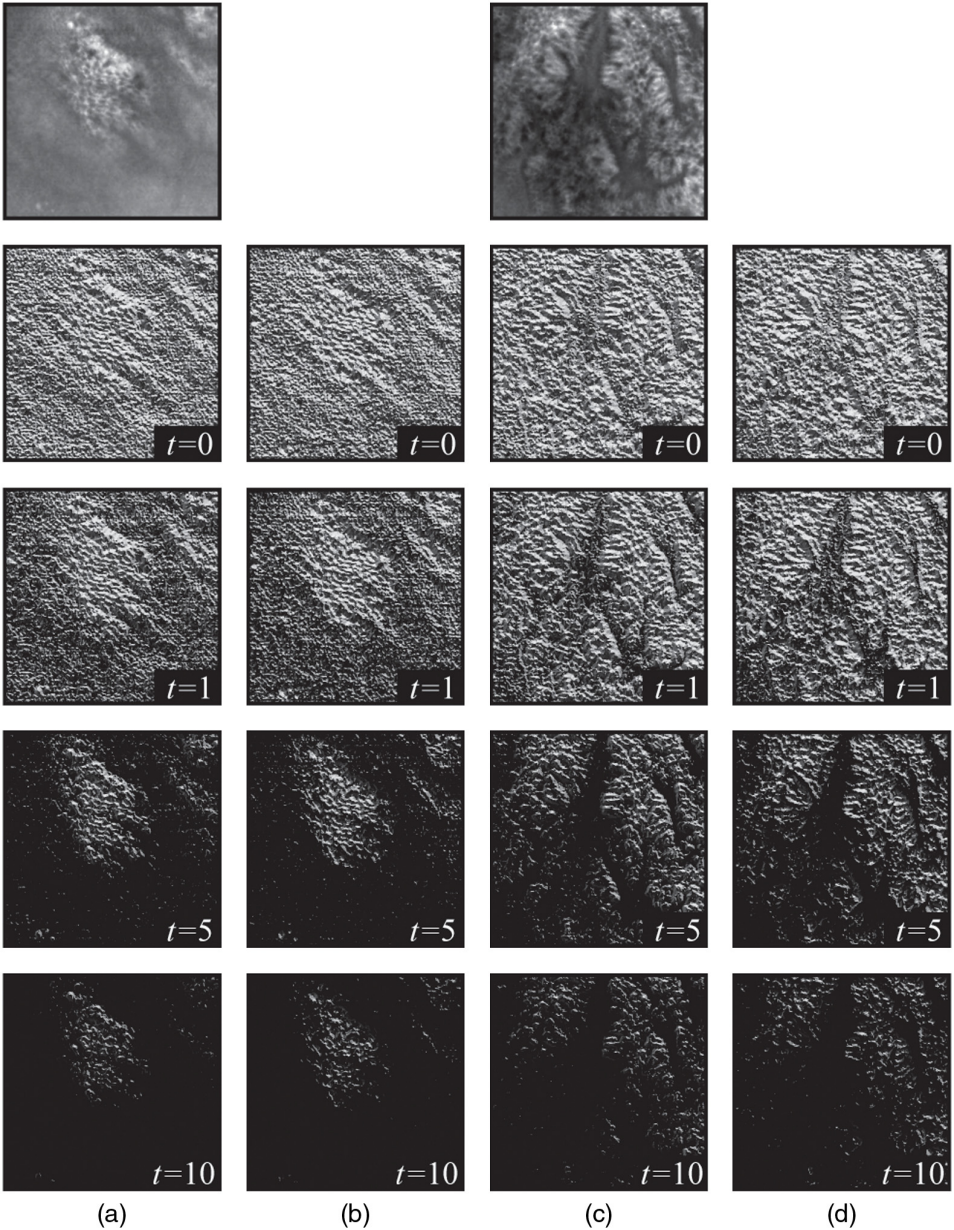
LTP of confocal endoscopy images at different thresholds, the thresholds of LTP are 0, 1, 5, and 10 from top to bottom. (a) and (c) Up pattern. (b) and (d) Low pattern.

The obtained LTP map contains $3^{8}$ patterns when P=8 because LTP codes $C=T(g_{i}-g_{c})$ can be −1, 0, and 1. For lower computational complexity, the original LTP map is converted to an up-pattern and low-pattern map. The up-pattern map is obtained by turning the LTP code C from −1 to 0 in Eq. (10), and a low-pattern map is obtained by turning the LTP code C from 1 to 0 and −1 to 1. Therefore, the up-pattern and low-pattern maps both contain $2^{8}$ patterns with values ranging from 0 to 255 as LBP. Figure 2 shows the LTP map of the two confocal endoscopy images with different MOSs. Note that because of the threshold in the threshold function, LTP emphasizes the high variation region and underestimates the low variation region, which is similar to DE, while the choice of threshold value affects the screening strength of local variations. However, Fig. 2 shows that the LTP map retains an image structure like LBP; thus, LTP can describe image quality degradation. Considering that LTP captures the local variation before extracting the structure, we apply Weber’s law to the threshold function of LTP as follows:

$$T_{\mathrm{WB}}(g_{i}-g_{c})=\{\begin{array}{cc} 1, & \frac{g_{i}-g_{c}}{g_{c}}>t\\ 0, & \frac{\left|g_{i}-g_{c}\right|}{g_{c}}\leq t\\ -1, & \frac{g_{i}-g_{c}}{g_{c}}<-t \end{array},$$ (11)

where $T_{\mathrm{WB}}(g_{i}-g_{c})$ consults the form of Weber’s law regarding $g_{i}-g_{c}$ as ΔI, $g_{c}$ as I, and t as threshold k. To prevent the instability of the results, the overall image I is added by one. After introducing Weber’s law in the threshold function, the judgment of local variation is under the HVS.

Threshold t in the threshold function of LTP determines the ability to capture an image variation and further affects the image description. Therefore, the threshold t plays a key role in the performance of WB-LTP. Referring the form of Weber’s law, the threshold t is calculated as follows:

$$t=\frac{\tan(\text{mean}(I_{\mathrm{DE}}))}{256},$$ (12)

where $I_{\mathrm{DE}}$ is the DE map in Eq. (2), $\text{mean}(I_{\mathrm{DE}})$ denotes the average variation intensity of image I. Function tan() and factor 1/256 are used to transfer the range of t from [−π/2,π/2] to the range of $\frac{\Delta I}{I}$ for corresponding to Eq. (11). The threshold t indicates that the region above or below average variation intensity of the image will be regarded as the high variation or low variation region, respectively.

After LTP improvement, we conducted further analysis to enrich the information expression of images. Considering that the up and low channels denote different directions of patterns, we referred to the calculation of the gradient magnitude^14^ and computed the magnitude channel $I_{\mathrm{MAG}}$ as follows:

$$I_{\mathrm{MAG}}=\sqrt{I_{\mathrm{UP}}^{2}+I_{\mathrm{LOW}}^{2}}.$$ (13)

Because LTP reveals local structure distribution, it is useful to calculate the entropy of LTP to characterize image information as follows:

$$E=-\overset{255}{\underset{i=0}{\sum }}p_{i}\;\log_{2}\;p_{i},$$ (14)

where $p_{i}$ denotes the frequency of grayscale value i in the image. We calculated $E_{\text{up}}$, $E_{\text{low}}$, and $E_{\mathrm{MAG}}$. Finally, we obtained the WB-LTP feature $f_{\mathrm{WB}-\mathrm{LBP}}=\{h_{\text{up}},h_{\text{low}},h_{\mathrm{MAG}},E_{\text{up}},E_{\text{low}},E_{\mathrm{MAG}}\}$, where $h_{\text{up}}$, $h_{\text{low}}$, and $h_{\mathrm{MAG}}$ are the histograms of $I_{\text{up}}$, $I_{\text{low}}$, and $I_{\mathrm{MAG}}$, respectively.

### 2.4 Feature Extraction and Quality Model

**Fig. 3:**
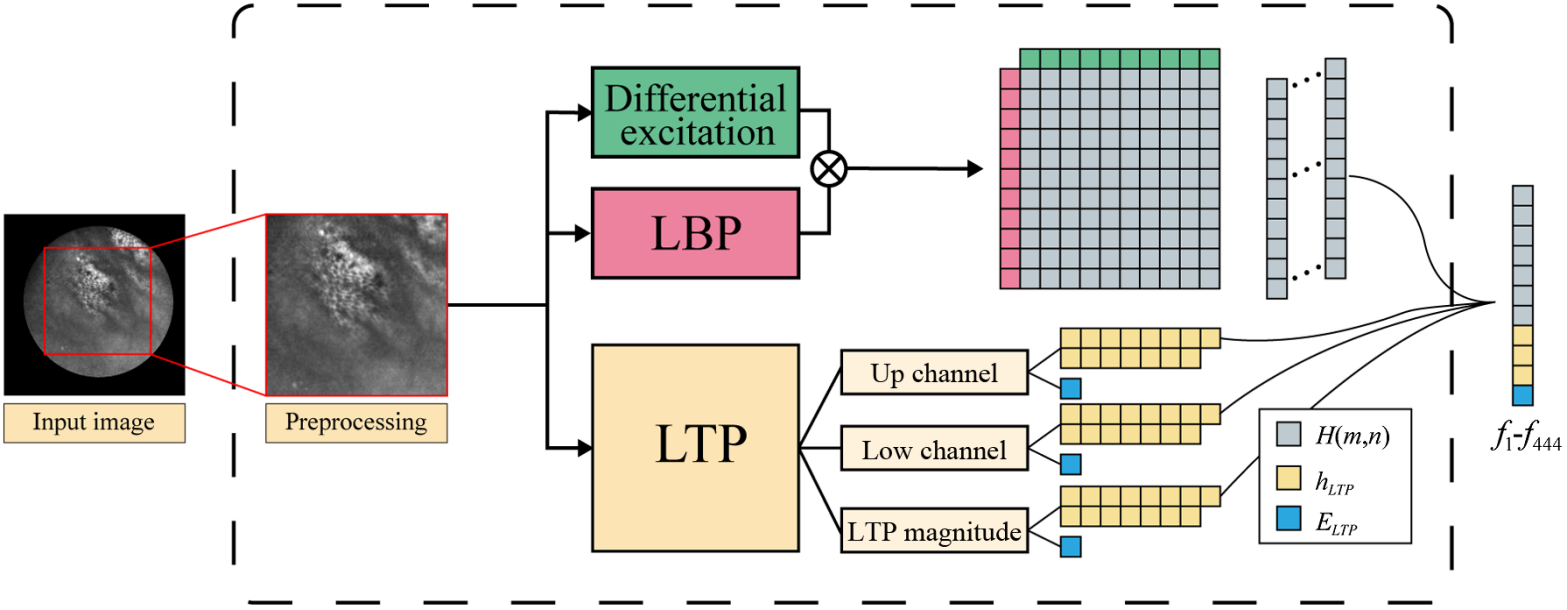
Flowchart of feature extraction. H(m,n) denotes DE-LBP feature, $h_{\mathrm{LTP}}$ and $E_{\mathrm{LTP}}$ denote WB-LTP feature.

Because the field-of-view of confocal endoscopy is circular, acquired images appear as circular effective regions surrounded by black regions, and the latter interferes with the image description. Therefore, before feature extraction, the image should be preprocessed by adopting the square inscribed in the valid circle region, as shown in Fig. 3. After preprocessing, the DE-LBP of the image was calculated using p=8 in DE, R=1, and P=8 in LBP and M=N=10 to obtain 100-dimensional features. Then, computing the WB-LTP of the image with 15 bins in the histogram and acquire 48 dimension features.

To obtain multiscale information of the image, the image is downsampled twice beside the origin scale. Features are extracted in three scales; thus, the features have 444 dimensions. In WB-LTP, the structure information differs from the three scales of images, leading to different thresholds. Therefore, the thresholds of different scales $t_{s}$ was calculated as $t_{s}=t/2^{s}$, where $1/2^{s}$ is the scale factor, s∈{0,1,2} is the number of image downsampling.

After obtaining the image features, SVR^43^ with a radial basis function kernel was adopted to build a quality prediction model.

### 2.5 Confocal Endoscopy Image Database

To compare the performance of IQA methods, we established a database of confocal endoscopy images. The imaging experiment was conducted using a confocal endoscope designed by Wang et al.^1^ The confocal endoscope has a field of view of 300×300  μm and a resolution of 4.4  μm, and the image was obtained with 1024×1024  pixels at a frame rate of 4 to 16 fps. Imaging experiments were conducted with *ex vivo* imaging of colonic and gastric tissues of female specific pathogen free and Sprague Dawley rats weighing ∼150  g. Imaging experiments obtained 656 images with blur, contrast distortion, and motion artifacts. All imaging experiments were approved by the animal experiment guidelines of the Animal Experimentation Ethics Committee of Huazhong University of Science and Technology (HUST, Wuhan, China).

To obtain meaningful MOS, eight researchers with extensive experience in instrument operation and image processing of confocal endoscopy rated the quality of images. Subjective quality assessment experiments apply single-stimulus (SS) methods. Every observer watches one confocal image 10 s on a computer monitor every time and gives a quality index ranging from one to five, where one denotes the lowest quality and five denotes the highest quality. To avoid observer exhaustion, a session lasted for half an hour and the observers watched 180 images. After a session, the observers rested for 8 min. Thus, there were four sessions in subjective quality assessment experiments.

**Fig. 4:**
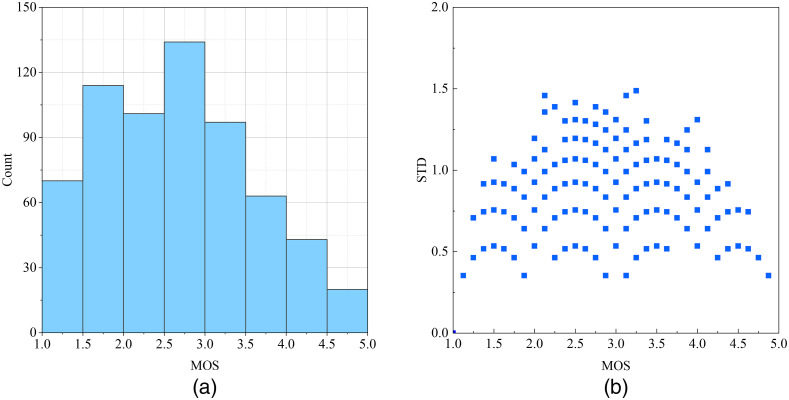
(a) MOS distribution of the established confocal endoscopy image database. (b) The relationship between MOS and corresponding STD of images in the confocal image database.

After obtaining the eight rates of every image, the standard deviation (STD) of every image quality score was calculated. The image with STD higher than 1.5 was discarded because the result cannot reflect objective image quality. Eight images were discarded in this process, and 642 images remained. Finally, the MOS of the images was computed by averaging the scores of the researchers, and the distribution of the image quality scores is shown in Fig. 4(a). The relationship between STD and MOS is shown in Fig. 4(b), which we can see that the consistency of observers’ opinions is higher when faced with relatively high-quality and low-quality images compared with images of medium quality.

## 3 Results

### 3.1 Experimental Protocol

We compared the proposed method with 11 state-of-the-art NR-IQAs with publicly available source codes. The methods used for comparison were the NSS feature-based IQA methods including BRISQUE^12^ and SINQ^44^ in the spatial domain, NBIQA^13^ in the spatial and DCT domains, and CurveletQA^16^ in the curvelet domain. There are local descriptor-based IQA methods including GWH-GLBP^20^ using a gradient magnitude-weighted LBP feature, ORACLE^45^ using the FAST algorithm, and the fast RetinaKeypoint descriptor (FREAK), NOREQI^23^ using the SURF algorithm, and RATER^46^ using FAST. In addition, there are image spatial and spectral entropy feature-based method SSEQ,^15^ free-energy feature-based NFERM,^18^ and gradient magnitude feature-based GM-LOG.^14^

All the above methods follow the process of feature extraction, SVR training, and prediction. Before feature extraction, all IQA models apply the same preprocessing method. For IQA employing color space information, grayscale values were used as inputs to the three color channels.

In the performance experiment, a confocal endoscopy image dataset was applied. First, 80% of the dataset were randomly chosen to train the SVR model, and the rest were used to test the performance. During the training phase of all IQA methods, SVR parameters were optimized using a grid search to achieve the best performance for fair comparison.

In the performance evaluation, Spearman rank order correlation coefficient (SROCC), Pearson’s linear correlation coefficient (PLCC), and root mean square error (RMSE) were used to characterize the monotonicity and accuracy of prediction. SROCC and PLCC values closer to 1 and RMSE closer to 0 indicates better performance. Before the PLCC and RMSE are calculated, the nonlinear logistic regression shown in Eq. (15) is required,^47^ where x is the predicted score, f(x) is the fitting score, and $\beta_{1-5}$ is the regression parameter:

$$f(x)=\beta_{1}(\frac{1}{2}-\frac{1}{\exp(\beta_{2}(x-\beta_{3}))})+\beta_{4}x+\beta_{5}.$$ (15)

The random 80% to 20% train-test is repeated 1000 times, and the median of performance criteria are reported. For a fair comparison, all methods use the same training and testing sets in each repeat.

### 3.2 Performance Comparison

Table 1 shows the performance of NR-IQA, and the best method is shown in bold. As shown in Table 1, CEIQA outperforms the other IQAs in all criteria, followed by NOREQI.

**Table 1:** Performance comparison of NR-IQA methods. The best IQA methods are highlighted in boldface.

IQA	SROCC	STD	PLCC	STD	RMSE	STD	Time (s)
BRISQUE^12^	0.8002	0.0346	0.8194	0.0301	0.5263	0.0343	0.0454
SSEQ ^15^	0.7324	0.0409	0.7510	0.0370	0.6042	0.0372	0.6583
CurvletQA^16^	0.7837	0.0361	0.7970	0.0319	0.5541	0.0344	1.3147
SINQ^44^	0.8094	0.0320	0.8249	0.0270	0.5175	0.0322	1.8766
NBIQA^13^	0.8017	0.0328	0.8175	0.0281	0.5292	0.0326	9.5719
GWH-GLBP^20^	0.8007	0.0305	0.8047	0.0280	0.5438	0.0335	0.0645
ORACLE^45^	0.8054	0.0307	0.8185	0.0273	0.5248	0.0310	0.3492
NOREQI^23^	0.8259	0.0286	0.8370	0.0250	0.5017	0.0304	0.3310
RATER^46^	0.6463	0.0476	0.6778	0.0429	0.6733	0.0350	11.8290
NFERM^18^	0.7948	0.0355	0.8137	0.0356	0.5309	0.0412	30.7871
GM-LOG^14^	0.7677	0.0367	0.7885	0.0327	0.5628	0.0355	**0.0441**
DE	0.7687	0.0379	0.7881	0.0347	0.5652	0.0374	0.0082
LBP	0.8286	0.0279	0.8454	0.0238	0.4916	0.0301	0.0696
DE-LBP	0.8401	0.0287	0.8552	0.0248	0.4771	0.0329	0.0799
LTP	0.8383	0.0282	0.8522	0.0235	0.4803	0.0292	0.0408
WB-LTP	0.8434	0.0253	0.8541	0.0217	0.4769	0.0288	0.0405
CEIQA	**0.8543**	**0.0251**	**0.8648**	**0.0215**	**0.4615**	**0.0283**	0.1166

**Fig. 5:**
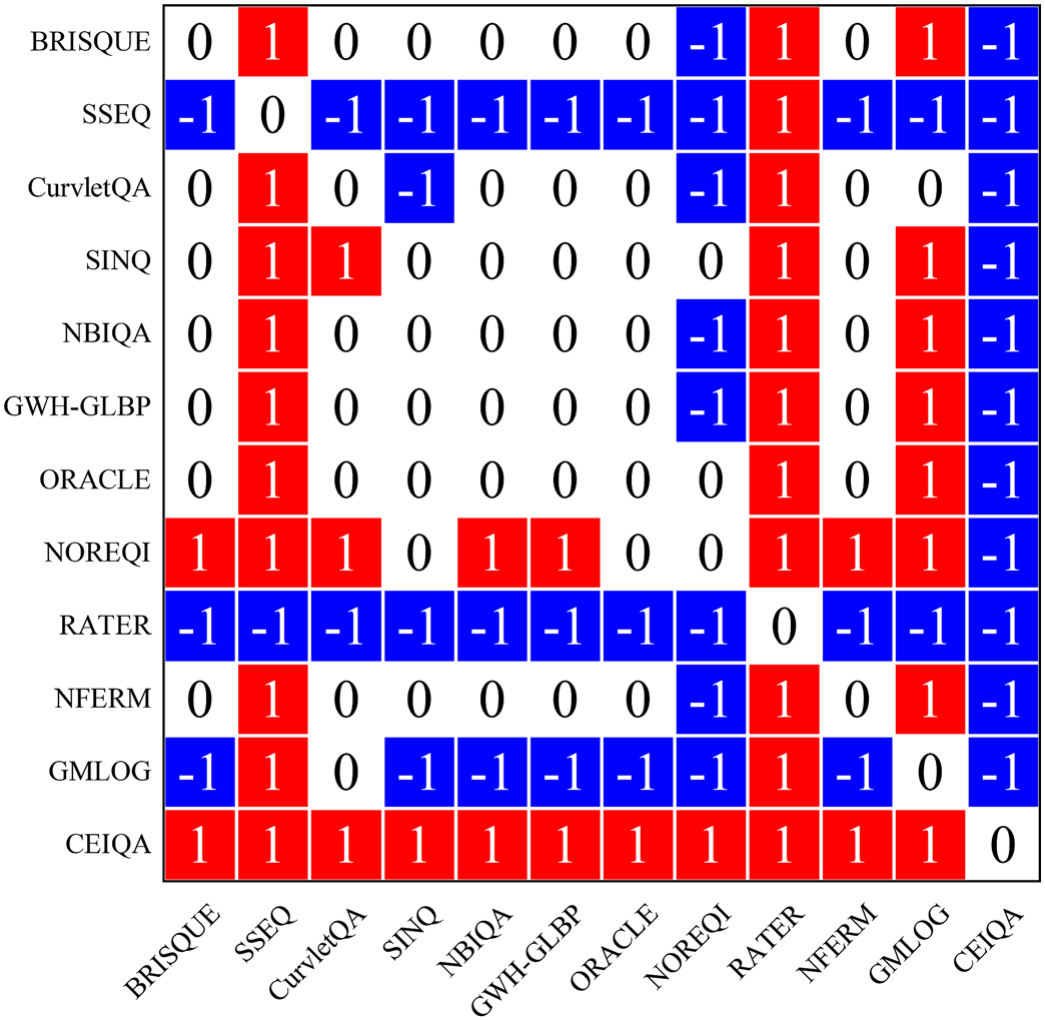
Results of statistically significant experiments using corrected resampled paired Student’s t-test. Symbols “1,” “−1,” and “0” mean that the method in the row is statistically (with 95% confidence) better, worse, or similar than the method in the column, respectively.

To further verify whether the performance difference is significant, we conducted the corrected resampled paired Student’s t-test^48^ between different methods of SROCC values of 1000 train-test repeats. The results are shown in Fig. 5, where symbols “1,” “−1,” and “0” mean that the method in the row is statistically (with 95% confidence) better, worse, or similar to the method in the column, respectively. Figure 5 shows that CEIQA is significantly superior to all other NR-IQA methods on the confocal endoscopy image dataset, followed by NOREQI.

**Fig. 6:**
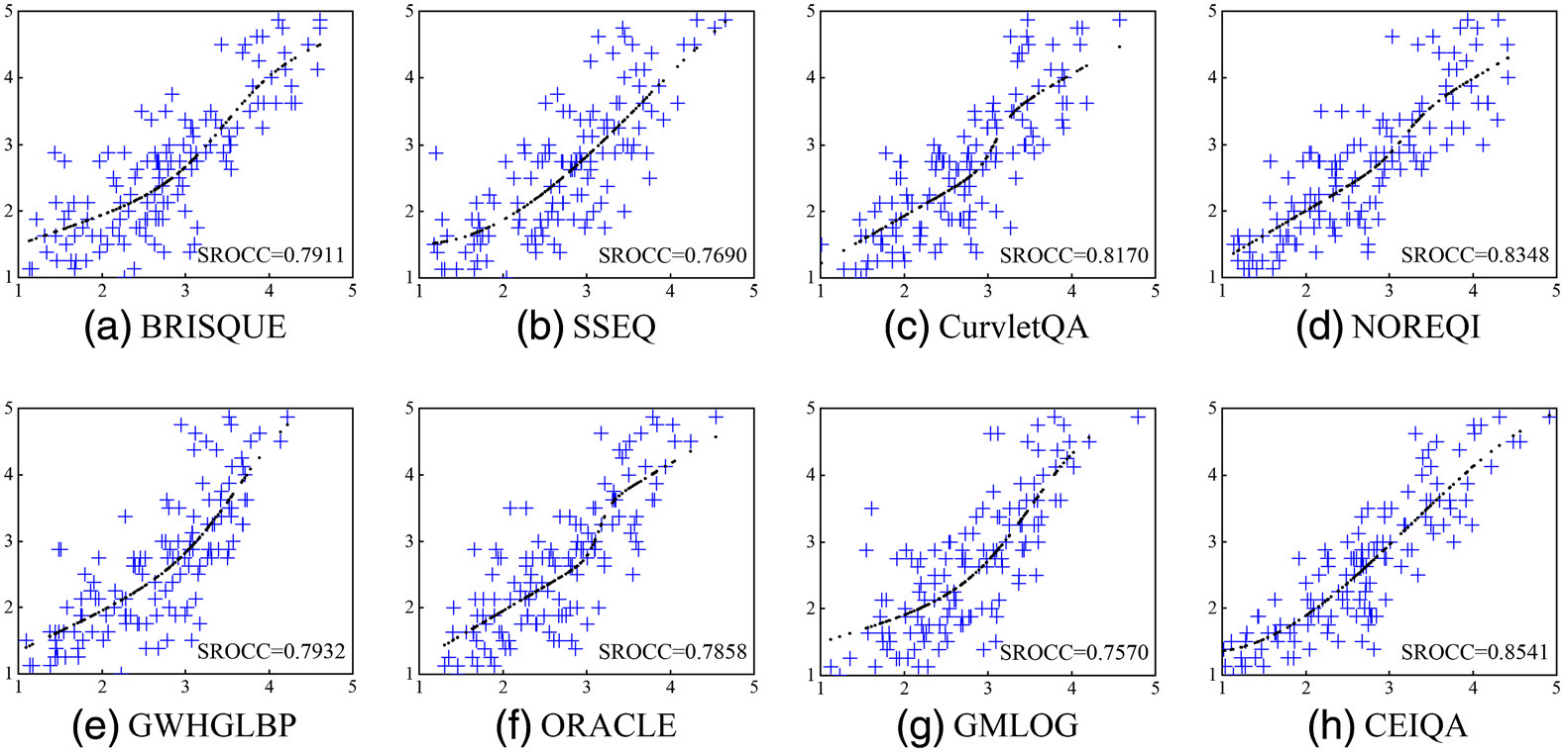
Scatter plots of MOS against the predicted MOS from the different IQA methods. The x axis denotes the predicted score of IQA methods, and the y axis denotes the MOS. The SROCC is reported in figures. The dashed line is the fitting curve calculated by Eq. (15).

To further analyze the relationship between the algorithm’s performance and the consistency between the predicted quality scores and MOS, the scatter plots of MOS against the predicted quality scores of the test dataset from the IQA methods in a train-test repeat are shown in Fig. 6. The corresponding SROCC values were labeled in the plots. For a clear view, we only show the results of BRISQUE, SSEQ, CurvletQA, NOREQI, GWHGLBP, ORACLE, GMLOG, and the proposed CEIQA method.

As shown in Fig. 6, the CEIQA with the highest SROCC shows the promising performance with most densely and evenly distribution of points around the fitting curve, which indicates the great consistency between MOS and the predicted quality scores. From Fig. 6, we can also see that the higher SROCC indicates better consistency between MOS and the quality scores predicted by the method.

The robustness of the algorithm is an important factor in the performance. To evaluate the robustness of IQA, we calculated the STD of the three criteria across 1000 repeats, which are shown in Table 1. The lower the STD, the more stable the algorithm performs for varying images. According to Table 1, CEIQA is the most stable among the IQA methods.

The rightmost column of Table 1 shows the time taken by different algorithms to extract the confocal endoscopy image features, which accounts for most of the total algorithm runtime. CEIQA has a promising speed and runs faster than NOREQI with the second-best performance. Experiments were performed on MATLAB R2017a with an Intel i7-8750HQ CPU at 2.20 GHz.

The experimental results demonstrated that CEIQA with a combination of perceptual laws and local descriptors has promising performance. Furthermore, to verify the enhancement of the proposed method using perceptual laws and the performance of the IQA components, we compared the performance of LBP and LTP before and after being improved using perceptual laws using the same 1000 train-test procedure. The results are presented in Table 1.

According to the results, the performance of LBP and LTP is remarkable, which demonstrates that the local descriptor is suitable for the quality prediction of confocal endoscopy images with multiple distortions. The same conclusion can be drawn from the fact that the NOREQI with the second-best performance also uses the local descriptors SURF, as shown in Table 1. Note that LTP uses the threshold calculation method proposed by Freitas et al.^49^ Furthermore, DE-LBP, which combines LBP and DE, significantly outperforms origin LBP, while WB-LTP also performs better than origin LTP owing to Weber’s law engagement. Introducing perceptual laws enhances the capability to describe image information and assess image quality.

### 3.3 Analysis of Parameters

**Fig. 7:**
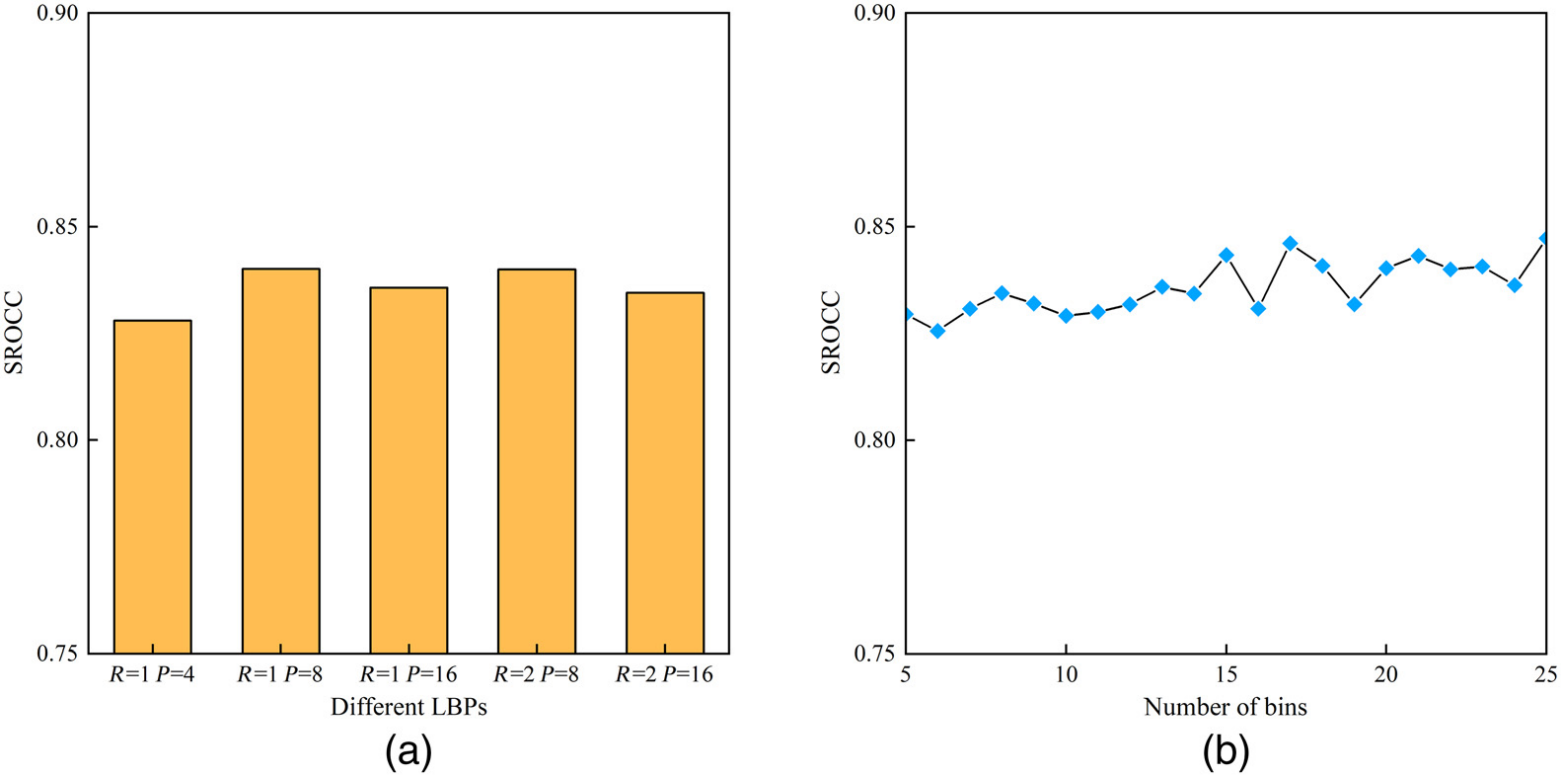
Performance of the proposed methods of different parameters. (a) SROCC of DE-LBP in different radius R and number of neighboring pixels P. (b) SROCC of different numbers of bins in WB-LTP histogram.

There are two types of parameters in the proposed CEIQA method. The first is the neighborhood radius R and the number of neighborhood pixels P in DE-LBP. The second is the number of bins in the WB-LTP histogram. To verify if the performance of CEIQA is sensitive to the variations of parameters, we performed two experiments with a variety of parameters. First, we compared the performance of DE-LBP for different values of R and P. The 1000 train-test process, as in Sec. 3.1, is conducted, and the median of the SROCC of the entire loop is shown in Fig. 7(a). Then, the performance experiment of WB-LTP for different numbers of bins was conducted, and the result is shown in Fig. 7(b).

As shown in Fig. 7, DE-LBP’s performance is stable across different settings of LBP and WB-LTP’s performance is stable in different bins number of the histogram in a moderately large range. In conclusion, the CEIQA, that consists of DE-LBP and WB-LTP, is robust to parameter variations and has good generalization capability.

## 4 Discussion

It is meaningful to analyze the relationship between the algorithm’s performance in predicting MOS and screening images. In clinical practice, image screening was employed by setting the quality threshold first, and then the images with a quality score lower than the quality threshold are labeled as “the low-quality image” which should be screened out and the images with a quality score higher than or equal to the quality threshold are labeled as “the high-quality image” that need to be kept. Therefore, the high consistency of the predicted quality scores and MOS indicates the high accuracy of image filtering. As shown in Table 1 and Fig 6, the proposed CEIQA method with great consistency between the predicted quality scores and MOS has the potential for practical application.

This study has some limitations. First, the confocal endoscopy images were obtained from a single confocal endoscopy instrument and two tissue types. The type of distortion is limited. This also limits the ability of the proposed CEIQA method to effectively characterize image quality. The lack of image morphology can cause the overfitting of the IQA. For example, IQA will provide higher quality scores to images with the tissue of high local variability and ignore distortion during imaging. The algorithm also must be improved to ignore image content and focus on image distortion. Second, the objectivity of subjective IQA of image MOS must be further improved, which in turn will affect the performance evaluation and application potential of the algorithm.

Future work will focus on obtaining confocal endoscopy images of more tissues, imaging conditions, and more detailed and clear types of distortion, as well as conducting more comprehensive subjective IQA experiments to obtain more meaningful MOS. With additional data, CEIQA is expected to show better performance and can be universally applied. Other directions of research could involve using the IQA method to evaluate and improve confocal endoscopy image enhancement and deconvolution algorithms or using the IQA method to select high-quality images in clinical practice and analyzing the effectiveness of the method.

## 5 Conclusion

In this study, we proposed a new NR-IQA method named CEIQA based on Weber’s law and a local descriptor. The image structural information is measured using the local descriptor, which is then improved by Weber’s law to extract perceptual features. The method is compared with 11 state-of-the-art NR-IQA methods on the introduced dataset of confocal endoscopy images. The dataset contains 642 images with authentic distortion and the corresponding MOS assessed by eight experimenters. As shown in the experimental results, CEIQA is significantly superior to other NR methods in terms of accuracy and robustness, which demonstrates that CEIQA has great potential for practical application and contributes to clinical diagnosis.

## Code, Data, and Materials Availability

The confocal endoscopy image dataset can be obtained from the authors upon reasonable request. The CEIQA source code and demo code is available in the Figshare repository via the link https://figshare.com/articles/software/CEIQAcode/16987909.
